# IUC24411-77 In search of metastasis-initiating cells in prostate cancer

**DOI:** 10.1093/oncolo/oyaf248.012

**Published:** 2025-08-29

**Authors:** Julia Richert, Robert Wenta, Anna Muchlińska, Beata Pieczyńska-Uziębło, Anna Żaczek, Kevin Miszewski, Marcin Matuszewski, Natalia Bednarz-Knoll

**Affiliations:** Laboratory of Translational Oncology, Medical University of Gdańsk, Poland; Laboratory of Translational Oncology, Medical University of Gdańsk, Poland; Laboratory of Translational Oncology, Medical University of Gdańsk, Poland; Laboratory of Translational Oncology, Medical University of Gdańsk, Poland; Laboratory of Translational Oncology, Medical University of Gdańsk, Poland; Department of Urology, Medical University of Gdańsk, Poland; Department of Urology, Medical University of Gdańsk, Poland; Laboratory of Translational Oncology, Medical University of Gdańsk, Poland

## Abstract

**Background:**

This study aimed to define the extent and complexity of tumor dissemination in patients with d’Amico high-risk prostate cancer (PCa), with a focus on identifying putative metastasis-initiating cells.

**Methods:**

Tumor-draining vein blood (TDVB) and peripheral blood (PB) samples were collected from 118 patients with high-risk localized PCa undergoing radical prostatectomy, alongside PB from 16 patients with newly diagnosed metastatic PCa. Circulating tumor cells (CTCs) were enumerated, phenotyped, and morphologically analyzed using 2 immunofluorescence protocols: (1) epithelial/EMT protocol (pan-keratin/vimentin/DAPI/CD45/CD31/αSMA/CD29), capturing general dissemination and (2) EGFR/AR protocol (pan-keratin/EGFR/AR/DAPI/CD45/CD31), focused on identifying metastasis-related and druggable CTC phenotypes. Imaging flow cytometry enabled single-cell image acquisition. Statistical analyses were conducted in IBM SPSS Statistics, and representative images were shared via CTC Atlas.

**Results:**

Over 2,500 CTCs were analyzed. The epithelial/EMT protocol defined 4 major CTC phenotypes (K^+^V^-^, K^+^V^+^, K^-^V^+^, K^-^V^-^), each exhibiting notable morphological diversity. CTCs were more abundant in TDVB, primarily of epithelial phenotype, while EMT-like, smaller CTCs were enriched in PB and associated with shorter time to biochemical recurrence. Platelet-coated epithelial CTCs in TDVB correlated with high serum TGF-β levels and disease progression, suggesting platelet-mediated modulation of CTC phenotype and metastatic potential. The EGFR/AR protocol identified a rare EGFR^high^/AR^neg^ CTC population associated with aggressive disease. This phenotype was also observed in matched tumor tissue and bone metastases and was characterized by dedifferentiation, disrupted protein trafficking, and impaired vascularization. EGFR^high^/AR^neg^ CTCs were enriched after androgen deprivation therapy and showed potential tropism for bone, potentially linked to tissue stiffness.

**Conclusions:**

Dual CTC phenotyping reveals distinct patterns of tumor dissemination and identifies a metastasis-associated EGFR^high^/AR^neg^ CTC phenotype with clinical relevance. These findings contribute to a deeper understanding of CTC biology and support the use of phenotypic profiling for patient stratification and treatment guidance in PCa.

**Funding:**

This work was funded by National Science Center (#2017/26/D/NZ5/01088, #2020/39/B/NZ5/01258) and Ministry of Science and Higher Education, Poland (#NdS-II/SP/0398/2023/01). See www.CTCAtlas.org for related data.

